# Physical activity status by pain severity in patients with knee osteoarthritis: a nationwide study in Korea

**DOI:** 10.1186/s12891-018-2301-6

**Published:** 2018-10-20

**Authors:** Hye-Young Shim, Mira Park, Hee-June Kim, Hee-Soo Kyung, Ji-Yeon Shin

**Affiliations:** 1 Department of Preventive Medicine, School of Medicine, Eulji University, Deajeon, Republic of Korea; 2 Department of Rehabilitation Medicine, Seoul National University Bundang Hospital, Seoul National University College of Medicine, Seongnam, Republic of Korea; 3 Department of Orthopaedic Surgery, Kyungpook National University Hospital, Deagu, Republic of Korea; 4 Department of Preventive Medicine, School of Medicine, Kyungpook National University, Daegu, Republic of Korea

**Keywords:** Osteoarthritis, Knee, Physical activity, Exercises, Pain

## Abstract

**Backgrounds:**

Few reports have explored the extent to which physical activity is affected by pain severity in knee osteoarthritis (KOA) patients. We used national representative data to investigate the physical activity of KOA patients compared to the general population to determine what proportion of patients met physical activity recommendations and to explore how the proportion changes with pain severity.

**Methods:**

We used data from the fifth Korean National Health and Nutrition Examination Survey (KNHANES V; 2010–2012). In total, 1279 participants aged ≥50 years who had radiographic KOA and who evaluated knee pain on a numerical rating scale were selected. KOA was assessed using the Kellgren–Lawrence system. The Korean short version of the International Physical Activity Questionnaire was used to measure physical activity status. We used the physical activity recommendations of the American College of Rheumatology Work Group Panel when evaluating the extent of activity in KOA patients.

**Results:**

Only 18.6% of KOA patients met the osteoarthritis expert panel recommendations, lower than in the general population (23.2%; *p* = 0.003). The percentages that met the recommendations in the none to mild pain group, moderate pain group, and severe pain group were 23.4%, 17.6%, and 18.3%, respectively (*p* = 0.341). In terms of flexibility, a somewhat higher percentage of those with moderate pain engaged in physical activity compared to those with little or no pain (17.1% vs. 12.3%), but the difference was not significant (*p* = 0.585).

**Conclusions:**

Regardless of pain severity, overall physical activity was suboptimal in Korean KOA patients. It is important to emphasize to osteoarthritis patients in clinical settings the need for physical activity, and a policy-based effort is required to facilitate appropriate exercise.

**Electronic supplementary material:**

The online version of this article (10.1186/s12891-018-2301-6) contains supplementary material, which is available to authorized users.

## Background

Knee osteoarthritis (KOA) is a degenerative joint disease that is common in the elderly; however, it also affects younger people [1]. KOA symptoms can limit physical activity and cause debilitating pain [1]. The World Health Organization (WHO) estimates that around 13–15% of adults aged over 55 years have KOA worldwide [2]. In Korea, because of the rapid aging of the population, it is expected that the burden of disease caused by osteoarthritis will increase, and care and management are thus becoming increasingly important [3]. If KOA symptoms lead to decreased mobility of the patients, then, patients can be more dependent on others and their quality of life can be compromised [4, 5]. The goal of KOA management is to improve quality of life and physical function, thereby, minimizing disability in daily life.

Considerable evidence suggests that physical activity can improve physical function [6, 7], reduce pain, and improve patient-reported disabilities [6, 7]. Currently, several different guidelines emphasize the importance of physical activity. The Osteoarthritis Research Society International (OARSI) document [8] recommends land- or water-based exercise and strength training as appropriate, and the American Academy of Orthopedic Surgeons (AAOS) [9] recommends that patients with symptomatic KOA engage in self-management programs; perform strengthening, low-impact aerobic exercises; and engage in physical activity consistent with the national guidelines. The American College of Rheumatology Work Group Panel [10] proposed that KOA patients perform 30 min of moderate-intensity (50–70% maximal heart rate) exercise 3 days a week. In addition, the American Geriatrics Society Panel on Exercise and Osteoarthritis [11] recommended engaging in muscle-strengthening activity 2–3 days/week and in flexibility activity 3–5 days/week. In Korea, exercise guidelines [12] recommend low-impact aerobic exercise.

However, there have not been enough studies that examined whether KOA patients engage in appropriate physical activity, especially in East Asia, although several Western studies have reported that the physical activity rates were suboptimal [13]. Moreover, recent studies have shown that osteoarthritis patients are at a higher risk for cardiovascular disease and death because of insufficient physical activity [14]. Thus, from the perspectives of both public health and geriatrics it is important for patients with osteoarthritis to maintain an appropriate level of physical activity.

Pain is one of the major symptoms of osteoarthritis [15]. The extent of pain is associated with decreased physical activity [15]. However, few reports have explored the extent to which physical activity is affected by pain severity in KOA patients.

The populations of Korea and other East Asian countries are aging rapidly, and their body mass indices [16] and lifestyles [17] differ from those of Western populations. Therefore, more evidence with respect to physical activity status of KOA patients in East Asia is needed.

In this study, we aimed to 1) investigate the levels of physical activity among KOA patients compared to the general population, 2) determine the proportion of KOA patients who meet the physical activity recommendations, and 3) examine how the proportion changes with pain severity among KOA patients, by using data from a nationally representative Korean population.

## Methods

### Data sources

We used data from the fifth Korea National Health and Nutrition Examination Survey (KNHANES V; 2010–2012); KNHANES is an ongoing, multicomponent, nationally representative survey of the noninstitutionalized Korean population administered by the Korea Centers for Disease Control and Prevention (KCDC). The survey uses a multi-stage clustered probability design, creating sampling units from household registries that vary by sex, region, and age group. The KNHANES V (2010–2012) survey evaluated a total of 576 primary sampling units and 11,520 households from approximately 200,000 geographically defined primary sampling units for the whole country over 3 years [18]. Each KNHANES assessment consists of a health interview, a health examination, and a nutrition survey. We extracted data from the health interview and health examination; we included sociodemographic factors, physical activity parameters, details of morbidities, and radiographic findings. The details of the survey methods and contents have been described elsewhere [18, 19]. The survey was approved by the institutional review board (IRB) of the KCDC in 2010–2012 (approval nos. 2010-02CON-21C, 2011-02CON-06-C, and 2012-01EXP-01-2C).

### Radiographic examination of the knee and definition of KOA

In KNHANES 2010–2012, osteoarthritic radiological examinations were performed on those aged ≥50 years. Of the 25,534 individuals who participated, 9514 individuals age > 50 years were subjected to radiographic examination of the knee joints in mobile examination cars based in four different provinces. All examinations were performed by four trained radiologists using digital X-ray machines (SD3000 Synchro Stand; SYFM, Namyangju, South Korea). Bilateral anterior-posterior, lateral (30° flexion), and weight-bearing anterior-posterior plain radiographs of the knees were taken. Two radiologists performed individual radiographic evaluations referencing the Kellgren–Lawrence grading system (0 = normal, 1 = suspicious, 2 = mild osteoarthritis, 3 = moderate osteoarthritis, and 4 = severe osteoarthritis) [20]. We defined KOA of Kellgren–Lawrence grade ≥ 2 as radiographic KOA.

To ensure the reliability and validity of osteoarthritis examination, quality control was conducted through 1) professional surveyor education, 2) equipment quality control, and 3) quality control of the radiograph reading system.

Concerning surveyor education, a site survey management manual was developed and directed to the professional surveyors. Before the start of the osteoarthritis examination, the surveyors were educated about bone and joint digital radiography filming by using the manual. With respect to equipment quality control, an area was selected randomly each month among 192 survey districts and on-site visits were performed more than 20 times in a year. Regular inspection of measurement equipment was conducted once a year. Daily equipment inspection was conducted according to the inspection items designated by the professional inspector on the day of the survey, and any problem with the equipment was immediately reported and addressed with corrective actions. The quality of X-ray imaging by different radiographers was assessed using the newly developed “Knee Joint Clinical Image Evaluation Form.” The average score for bone and joint radiograph quality was 87.76 out of 100. Concerning the quality control of the radiograph reading system, data from the osteoarthritis examination using the reading system was uploaded to and downloaded from Webhard, and graded after double reading by two radiologists, using the Kellgren–Lawrence grading system. In 2010 and 2011, the radiographic digital images were graded by two radiologists. In 2012, one of the two radiologists read all images, and 5% of the images were read by another radiologist. If the grades differed by more than two points, those digital data were read by another radiologist. Inter-rater and intra-rater reliabilities were assessed annually. The measurement methods and quality control procedures are described in detail elsewhere [21, 22].

### Pain inclusion criteria

Knee pain was assessed in those participants who complained of pain on > 30 days during the previous 3 months using the question “Please describe the average pain in the knee joint, regardless of the medication used? Please indicate this on a 0~10-point scale with higher scores representing greater pain severity.” The numerical rating scale (NRS) answers were divided into three groups (0–3 points = none to mild pain, 4–6 points = moderate pain, ≥7 points = severe pain) [23].

Of the 9514 participants aged ≥50 years who underwent radiographic examinations, 3483 had radiographic KOA. Of these, 1279 who had NRS data were included as the final study subjects.

### Physical activity

The KNHANES 2010–2012 physical activity questionnaire was based on the Korean short version of the International Physical Activity Questionnaire (IPAQ) [24]. This consists of six questions: the number of days on which vigorous physical activity was performed in the previous 7 days, and the usual duration of such activity; the number of days on which moderate physical activity was performed in the previous 7 days, and the usual duration of such activity; and the number of days on which the subject walked for at least 10 min at a time during the previous 7 days, and the usual duration of walking. The numbers of days on which muscle-strengthening and flexibility activities were performed during the previous 7 days was also noted.

Based on the short-form IPAQ responses, physical activity was divided into three categories [25]: inactive (Category 1), minimally active (Category 2), and health-enhancing physical activity (HEPA; Category 3). The inactive group (Category 1) reflected the lowest level of physical activity. Those who did not meet the criteria for Categories 2 or 3 were considered inactive. The minimally active group (Category 2) included those who engaged in a) ≥20 min of daily vigorous activity on ≥3 days, or b) ≥30 min of moderate-intensity activity or walking on ≥5 days, or c) any combination of walking and moderate- or vigorous-intensity activity on ≥5 days that summed to ≥600 MET-min/week. The HEPA group (Category 3) met either of the following criteria: a) vigorous-intensity activity at least 3 days summing to ≥1500 MET-min/week or b) any combination of walking and moderate- or vigorous-intensity activity that summed to ≥3000 MET-min/week. The methods for calculating activity are described in the IPAQ guidelines [25].

The American College of Rheumatology Work Group Panel has recommended physical activity or exercise at least 3 days a week (at 50–70% maximal heart rate) for KOA patients [10] (hereafter, the “OA expert panel recommendation”). We classified patients as meeting the recommendation or not meeting the recommendation. In addition, based on the recommendations of the American Geriatrics Society Panel on Exercise and Osteoarthritis [11], we categorized patients according to whether they met the recommendations in terms of muscle-strengthening and flexibility activity. The group that met the recommendation for muscle-strengthening activity engaged in such activity 2–3 days/week; those who did not meet the recommendation did so on ≤1 or ≥ 4 days/week. The group that met the recommendation for flexibility activity engaged in such activity 3–5 days/week; those who did not meet the recommendation did so on ≤2 or ≥ 6 days/week.

### Other characteristics of the participants

Age (50–59, 60–69, and ≥ 70 years), monthly household income (in quartiles), education level (less than or equal to elementary school, middle school, high school, and college or higher), and marital status (with or without spouse [separated, bereaved, divorced]) were the sociodemographic factors evaluated. Income per adult equivalent was calculated as household income divided by the square root of the number of persons in the household. Depressive mood was explored using the question “Have you ever felt sad or desperate over the past year for 2 consecutive weeks or more?” The possible responses were “yes” or “no.” Body mass index (BMI) was calculated as body weight divided by height squared (kg/m^2^); participants with BMI ≥25.0 kg/m^2^ were considered obese according to WHO criteria [26]. The number of comorbidities was the sum of diseases diagnosed by a doctor. Smoking status was categorized as smoker or nonsmoker. Alcohol consumption was categorized based on high-risk drinking (more than seven drinks at a time for males and five for females) and as never or low (< 1 episode/month of high-risk drinking), moderate (1–3 episodes/month), and excessive (≥4 episodes/month) drinking [27].

#### Statistical analysis

The chi-square test was used to compare the characteristics of KOA patients to those of the general population and also to analyze physical activity status by pain severity. Univariable and multivariable logistic regression was performed to identify factors affecting the inability to meet the recommendations of the osteoarthritis expert panel in terms of physical activity. Logistic regression yielded odds ratios (ORs) and 95% confidence intervals (CIs). A two-tailed *p* value < 0.05 was deemed statistically significant in all analyses. All analyses were performed using SAS ver. 9.4 (SAS Institute Inc., Cary, NC, USA).

## Results

Table 1 compares the general characteristics and physical activity of KOA patients with those of the general population older than 50 years of age. Of the 1279 KOA patients, 221 (16.5%) were males and 1058 (83.5%) were females. The percentage of females was higher among KOA patients (83.5%) than in the general population (49.7%). The percentage of low-income KOA patients was twice that in the general population (53.1% vs. 25.4%, respectively). KOA patients also had less education and a more depressive mood than the general population. In terms of physical activity as classified by the IPAQ, more KOA patients were inactive (61.1%) than in the general population (53.3%; *p* < 0.001). Only 18.6% of KOA patients met the OA expert panel recommendations, lower than in the general population (23.2%; *p* = 0.003). The proportions of KOA patients who met recommendations for muscle-strengthening and flexibility activity were 4.3% and 15.8%, significantly lower than in the general population (14.6% and 26.2%, respectively; *p* < 0.001).

**Table 1:** Comparison of the general characteristics and physical activity of knee osteoarthritis patients and the general population aged ≥50 years

	Knee osteoarthritis patients (≥50 years of age) (*N* = 1279)		General population (≥50 years of age) (*N* = 7917)		*P*-value
	*n*	Weighted %	*n*	Weighted %	
Sex					
Male	221	16.5%	3707	50.3%	< 0.001
Female	1058	83.5%	4210	49.7%	
Age					
50–59 years	154	15.1%	3226	52.3%	< 0.001
60–69 years	423	33.0%	2601	27.1%	
≥ 70 years	702	51.8%	2090	20.6%	
Monthly household income					
Low	677	53.1%	2189	25.4%	< 0.001
Moderate to low	290	23.5%	2053	26.2%	
Moderate to high	157	12.9%	1716	23.4%	
High	136	10.6%	1869	25.1%	
Education level					
≤ Elementary	1010	80.4%	3402	41.4%	< 0.001
Middle school	152	11.3%	1422	19.7%	
High school	92	6.8%	2033	26.5%	
≥ College	23	1.5%	1040	12.4%	
Marital status					
With spouse	504	42.5%	1427	17.8%	< 0.001
Without spouse	767	57.5%	6416	82.2%	
Depressive mood					
No	991	77.3%	6752	85.1%	< 0.001
Yes	279	22.7%	1128	14.9%	
Body mass index					
> 25 kg/m^2^	500	43.7%	4258	61.5%	< 0.001
≤ 25 kg/m^2^	620	56.3%	2582	38.5%	
Number of comorbidities					
0	360	28.7%	3141	42.6%	< 0.001
1	441	35.2%	2574	31.7%	
2	292	22.3%	1447	17.4%	
≥ 3	186	13.9%	755	8.3%	
Cigarette smoking					
Nonsmoker	1168	90.6%	6554	79.5%	< 0.001
Smoker	104	9.4%	1326	20.5%	
Alcohol consumption					
Never	700	54.1%	2957	34.2%	
Low	345	27.3%	2114	25.1%	< 0.001
Moderate	150	11.6%	1574	21.0%	
Excessive	77	7.0%	1232	19.7%	
IPAQ					
Inactive	752	61.1%	4200	53.3%	< 0.001
Minimally active	352	25.9%	2443	30.1%	
HEPA active	166	13.0%	1233	16.5%	
Muscle-strengthening activity (2–3 days/week)					
Not met	1219	95.7%	6793	85.4%	< 0.001
Met	60	4.3%	1124	14.6%	
Flexibility activity (3–5 days/week)					
Not met	1081	84.2%	5870	73.8%	< 0.001
Met	198	15.8%	2047	26.2%	
Osteoarthritis expert panel recommendation^a^					
Not met	1014	81.4%	6071	76.8%	0.003
Met	256	18.6%	1804	23.2%	

*IPAQ* International physical activity questionnaire, *HEPA* Health-enhancing physical activity

^a^Osteoarthritis expert panel recommendation: Performance of 30 min of moderate-intensity (50–70% maximal heart rate) physical activity or exercise at least 3 days a week

Table 2 shows physical activity status by pain severity among KOA patients. According to pain severity, 152 (11.9%), 434 (33.9%), and 693 (54.1%) patients had none to mild, moderate, and severe pain, respectively. Regardless of pain severity, overall physical activity was suboptimal. The level of physical activity did not differ significantly by pain severity. This was true for all types of physical activity, including IPAQ, muscle-strengthening, and flexibility activity. The proportions of minimally HEPA+ active patients by increasing pain severity were 47.4%, 38.5%, and 38.9%, respectively (*p* = 0.142). The proportions who met OA expert recommendations by increasing pain severity were 23.4%, 17.6%, and 18.3%, respectively (*p* = 0.341). Overall, the group with little or no pain engaged in slightly more physical activity than the group with severe pain, but the difference was not statistically significant. Those with moderate and severe pain exhibited little difference in physical activity. In terms of IPAQ, muscle-strengthening, and OA expert panel–recommended activity, the group with severe pain engaged in somewhat more physical activity than the group with moderate pain, but the difference was not statistically significant. In terms of flexibility activity, the group with little or no pain engaged in less activity than the group with moderate pain (12.3% vs. 17.1%), but again the difference was not statistically significant (*p* = 0.585; Table 2).

**Table 2:** Physical activity status by pain severity among knee osteoarthritis patients (*N* = 1279)

	Pain Severity						*P*-value
	None to mild (*n* = 152)		Moderate (*n* = 434)		Severe (*n* = 693)		
	*n*	Weighted %	*n*	Weighted %	*n*	Weighted %	
IPAQ^a^							
Inactive	80	52.6	244	61.6	428	61.1	0.142
Minimally active	47	31.1	130	27.9	175	25.9	
HEPA active	25	16.3	57	10.6	84	13.0	
Muscle-strengthening activity (2–3 days/week)^a^							
Not met	140	93.0	414	96.3	665	95.9	0.309
Met	12	7.0	20	3.7	28	4.1	
Flexibility activity (3–5 days/week)^a^							
Not met	133	87.7	355	82.9	593	84.3	0.585
Met	19	12.3	79	17.1	100	15.7	
Osteoarthritis expert panel recommendation^a,b^							
Not met	116	76.6	338	82.4	560	81.7	0.341
Met	35	23.4	93	17.6	128	18.3	

Pain severity was categorized using numerical rating scale: 0–3 = none to mild pain, 4–6 = moderate pain, and 7–10 = severe pain

*IPAQ* International physical activity questionnaire, *HEPA* Health-enhancing physical activity

^a^The totals do not equal 1279 because of missing data

^b^Osteoarthritis expert panel recommendation: Performance of 30 min of moderate-intensity (50–70% maximal heart rate) physical activity or exercise at least 3 days a week

We performed logistic regression analyses to identify factors affecting the inability of KOA patients to meet the OA expert recommendations. In univariable analyses, KOA patients aged 70 years or older were less likely to meet the recommendations (OR = 0.6, 95% CI = 0.38–0.96; Table 3) and patients with a spouse were more likely to meet the recommendations (OR = 1.6, 95% CI = 1.16–2.24; Table 3). Those who drank to excess were more likely to meet the recommendations (OR = 2.0, 95% CI = 1.17–3.52; Table 3). In multivariable analyses, however, the above three variables were no longer significant. Only > 3 comorbidities was associated with an inability to meet the recommendations (OR = 0.5, 95% CI = 0.27–0.94; Table 3).

**Table 3:** Factors associated with compliance with the exercise recommendations of experts on osteoarthritis^a^

	Univariable analyses			Multivariable analyses^b^		
	OR	95% CI		OR	95% CI	
Sex						
Male	reference	–	–	reference	–	–
Female	0.8	0.57	1.24	1.0	0.55	1.64
Age						
50–59 years	reference	–	–	reference	–	–
60–69 years	1.0	0.62	1.60	1.0	0.61	1.77
≥ 70 years	0.6	0.38	0.96	0.7	0.38	1.20
Monthly household income						
Low	reference	–	–	reference	–	–
Low to moderate	1.2	0.86	1.80	1.2	0.76	1.77
Moderate to high	0.8	0.47	1.35	0.6	0.34	1.14
High	1.3	0.80	2.11	1.1	0.61	1.90
Education level						
≤ Elementary school	reference	–	–	reference	–	–
Middle school	1.2	0.73	1.85	0.8	0.48	1.48
High school	1.3	0.76	2.30	1.2	0.64	2.33
≥ College	2.1	0.80	5.30	1.9	0.67	5.51
Marital status						
With spouse	reference	–	–	reference	–	–
Without spouse	1.6	1.16	2.24	1.3	0.90	2.00
Depressive mood						
No	reference			reference		
Yes	1.3	0.89	1.81	1.5	0.98	2.19
Body mass index						
> 25 kg/m^2^	reference	–	–	reference	–	–
≤ 25 kg/m^2^	1.1	0.79	1.54	1.0	0.71	1.46
Cigarette smoking						
Nonsmoker	reference	–	–	reference	–	–
Smoker	0.6	0.33	1.20	0.6	0.26	1.18
Alcohol consumption						
Never	reference	–	–	reference	–	–
Low	0.7	0.50	1.09	0.7	0.46	1.09
Moderate	1.6	1.05	2.53	1.3	0.78	2.27
Excessive	2.0	1.17	3.52	1.6	0.77	3.16
Knee pain severity						
None to mild	reference	–	–	reference	–	–
Moderate	1.0	0.63	1.69	0.9	0.53	1.59
Severe	0.8	0.51	1.34	0.8	0.48	1.39
Number of comorbidities						
0	reference	–	–	reference	–	–
1	0.9	0.61	1.31	0.9	0.59	1.37
2	0.9	0.60	1.38	0.8	0.50	1.29
≥ 3	0.6	0.34	1.01	0.5	0.27	0.94

Pain severity was categorized using numerical rating scale: 0–3 = none to mild pain, 4–6 = moderate pain, and 7–10 = severe pain

*OR* Odds ratio, *CI* Confidence interval

^a^Performance of 30 min of moderate-intensity (50–70% maximal heart rate) physical activity or exercise at least 3 days a week

^b^The model included sex, age, monthly household income, education level, marital status, depressed mood, body mass index, cigarette smoking, alcohol consumption, knee pain severity, and number of comorbidities

## Discussion

We measured the proportions of KOA patients who met physical activity recommendations and assessed physical activity status by pain severity. The percentage of KOA patients who met the physical activity guidelines of OA expert panel recommendation [10] was 18.6%, lower than in the general population. In addition, physical activity status did not differ significantly by pain level, being uniformly suboptimal.

Low levels of physical activity by osteoarthritis patients have been reported in previous studies [13, 23, 28, 29]. In a U.S.-based study [23], osteoarthritis patients were less likely than adults without arthritis to engage in recommended levels of physical activity as in our study. In the study, 32.3% of osteoarthritis patients met the OA expert panel recommendation, which was significantly lower than the proportion of 39.5% reported for the adults without arthritis [23]. One meta-analysis found that the proportion of osteoarthritis patients who met the recommendation of ≥150 min per week of moderate to vigorous physical activity (MVPA) in bouts of ≥10 min (the physical activity level recommended for general adults) was only 13% and that the proportion of those who met the recommendation of at least 10,000 steps per day (another popular physical activity recommendation) was 19% [13], which were suboptimal proportions, as our study showed.

Pain is reported to be one of the primary causes of reduced physical activity among osteoarthritis patients [30, 31], because pain can be experienced during the performance of an activity. Although the group with none to mild pain engaged in slightly more physical activity than did the other groups, we found no proportional decrease in physical activity by pain severity, regardless of the type of activity. Indeed, in terms of flexibility activity, the group with severe pain exercised more than the group with little or no pain. However, the proportions of patients who met the recommended physical activity guidelines were notably suboptimal, even in the group with little or no pain. In this group, the proportions satisfying the recommended physical activity, muscle-strengthening, and flexibility activity recommendations were only 23.4%, 7.0%, and 12.3%, respectively. Although studies on the extent of physical activity according to pain severity in KOA patients are rare, White et al. [32] reported results similar to ours in that physical activity status was not statistically different according to pain level. Their study also showed that pain level did not significantly affect the attainment of the recommended physical activity levels [32]. The study was conducted by dividing male and female patients, and the percentages of men meeting the guidelines were 10.9%, 8.8%, and 12.9% and those of women were 11.0%, 8.6%, and 6.7% in the no, mild, and moderate/severe pain groups, respectively, with no apparent statistically significant differences [32]. These findings suggest that pain is not a critical barrier to performing exercise in osteoarthritis patients.

We performed multivariable analyses to explore further whether factors other than pain were associated with KOA patients’ (non)fulfilment of exercise recommendations. Previous studies found that physical activity was reduced in patients of older age [33, 34], on lower incomes [33], who were less educated [33], who were obese [35], and who received little social support [29]. In our study, we found that only the number of comorbidities was statistically significant. The more the comorbidities, the less the physical activity, as also reported by Dunlop et al. [33]. We found no significant factors other than poor health (i.e., three or more comorbidities), which suggests that there may be other factors not investigated in the survey besides the well-known individual factors that affect the physical activity status of osteoarthritis patients.

Together, our results suggest that barriers other than pain may cause KOA patients not to meet physical activity recommendations. Several possibilities are apparent. First, KOA patients may receive insufficient education in clinical settings. Currently, no treatment completely cures osteoarthritis [32]. When KOA patients visit clinics, the principal aim of conservative treatment is to minimize pain and limitations of joint function using pharmacological or nonpharmacological approaches. Of nonpharmacological treatments, appropriate physical activity is reportedly effective in maintaining joint mobility and improving muscle strength [36], and all major arthritis guidelines recommend moderate exercise [8–10]. However, in clinical settings, patients may be inadequately informed about how physical activity would assist them, types of exercise, and how often they should exercise. In fact, when we additionally assessed the experience of receiving education about arthritis management in osteoarthritis patients, more than 90% of all patients answered that they had not received any relevant education (Additional file 1: Table S1). Various institutional problems may be at play, including exercise education fees, too many patients per doctor, and a prohibitive payment system. Further research is needed to explore how to emphasize the importance of physical activity and the provision of appropriate guidelines.

Second, it is possible that although patients may wish to exercise, the absence of specific exercise guidelines (on kinds of exercise or the intensity and duration of exercise) renders engaging in activity difficult in the presence of pain. In fact, no specific activity guidelines are available for KOA patients with different grades of pain [8–11]. A systematic analysis of practice guidelines targeting osteoarthritis patients found that the guidelines varied by research group, institute, and professional society, as well as over time, and were based on lower-quality evidence [37]. The specific type of activity; the intensity, amount, and frequency of activity; the initial extent of joint exercise; how such exercise should be gradually increased; the duration of rest periods; and protective equipment by pain level all need to be included in the guideline.

Our study has certain limitations. First, this was a cross-sectional study, and we thus cannot address cause-and-effect questions. Second, information bias may have been at play; we used self-reported data to obtain information on physical activity and other variables. Third, and related to the second limitation, we did not use activity monitors such as accelerometers or heart-rate meters to measure physical activity. Rather, we used the self-administered IPAQ. Thus, the recorded physical activity levels may have been less accurate than those of studies that used activity monitors as surrogate markers of physical activity [38]. However, the IPAQ is a valid measurement tool, as reliable and valid as activity monitors in comparisons performed in about 12 countries, and is used in the U.S. National Health Interview Survey and various surveys conducted by the World Health Organization [24]. Fourth, as the study investigated Korean patients, caution should be taken when generalizing our results to other races with different lifestyles and anthropometric characteristics. Moreover, as our study population comprised > 80% women, caution is needed when generalizing our results to the entire KOA population.

Despite these limitations, our study is meaningful in that we investigated the physical activities of radiographically diagnosed osteoarthritis patients nationwide, calculated the proportions of such patients who met physical activity recommendations in terms of various types of activity, and explored physical activity status by severity of pain.

## Conclusions

We found that only 18.6% of Korean KOA patients met the physical activity recommendations, which was significantly lower than the proportion of 23.3% reported for the general population. Regardless of pain severity, overall physical activity was suboptimal in KOA patients. The proportion of patients who met OA expert recommendations was 23.4%, 17.6%, and 18.3% in the none to mild, moderate, and severe pain groups, respectively. In the clinical setting, it is important to emphasize the need for physical activity to patients with osteoarthritis, and a policy-based effort is required to develop physical activity guidelines that reflect pain severity and facilitate the delivery of appropriate exercise.

## Additional file

Additional file 1: **Table S1.** Experience of arthritis education among knee osteoarthritis patients. (DOCX 14 kb)

## Abbreviations

AAOS: American Academy of Orthopedic Surgeons
CI: Confidence interval
HEPA: Health-enhancing physical activity
IPAQ: International Physical Activity Questionnaire
KCDC: Korea Centers for Disease Control and Prevention
KNHANES: Korea National Health and Nutrition Examination Survey
KOA: Knee osteoarthritis
NRS: Numerical rating scale
OARSI: Osteoarthritis Research Society International
OR: Odds ratio
WHO: World Health Organization
