# Supplementary material for: Physical activity status by pain severity in patients with knee osteoarthritis: a nationwide study in Korea
Source: BMC Musculoskelet Disord. 2018 Oct 20;19:380. doi: 10.1186/s12891-018-2301-6 (PMC6195748; doi:10.1186/s12891-018-2301-6)
Supplement: Supplementary file 1 — Table S1. Experience of arthritis education among knee osteoarthritis patients. (DOCX 14 kb) [file 12891_2018_2301_MOESM1_ESM.docx]

Table S1. Experience of arthritis education among knee osteoarthritis patients

| Experience of OA education | n | % |
| --- | --- | --- |
| No | 1,249 | 97.7 |
| Yes | 30 | 2.3 |
